# Metformin prevents DMH-induced colorectal cancer in diabetic rats by reversing the warburg effect

**DOI:** 10.1002/cam4.521

**Published:** 2015-09-17

**Authors:** Yanglei Jia, Zengyi Ma, Xiaofei Liu, Wenjing Zhou, Shan He, Xia Xu, Guijie Ren, Gang Xu, Keli Tian

**Affiliations:** 1 Department of Biochemistry and Molecular Biology, Shandong University School of MedicineJinan, Shandong, China; 2 Department of Gastroenterology, 456 Hospital of PLAJinan, Shandong, China

**Keywords:** Colorectal cancer, diabetes mellitus, glucose metabolism, metformin, warburg effect

## Abstract

Epidemiologic studies have shown that the treatment of diabetics with metformin reduced the risk of cancer-related mortality. Here, we investigated the chemopreventive effects of metformin on dimethylhydrazine (DMH)-induced colorectal carcinogenesis in diabetic SD rats following metformin treatment and the effect on Warburg effect involved in this process. Diabetic rat models were induced with high-fat feeding in combination with a low dose of Streptozotocin (STZ) and then induce colorectal cancer with a low dose of DMH. The formation of colorectal Aberrant crypt foci (ACF) and the incidence, number and size of the tumor were measured. The proliferation indices of colonic tissues were determined through Proliferating cell nuclear antigen (PCNA) immunostaining. Then detect the expression of PK and IDH in colonic tissues using immunohistochemistry and Western blot. The enzyme activities of HK and PDH in colonic tissues were measured. The growth and expression of PK and IDH and activity of HK and PDH in cell lines LoVo and HT-29 were measured after metformin treatment. The results showed that metformin treatment significantly inhibited the formation of ACF and tumors. The proliferation index of colonic tissues was significantly decreased following metformin treatment. In addition, metformin inhibited cell growth and decreased the imbalance in the expression of the enzymes involved in glycolysis and the TCA cycle. These findings suggested that metformin might produce a synergistic colon cancer-preventative effect in diabetic patients through the regulation of the enzymes expression involved in glucose metabolism.

## Introduction

Diabetes mellitus and colorectal cancer are two common and frequently occurring diseases 1,2. Epidemiologic studies have shown that type 2 diabetes mellitus is closely associated with the increased risk of colorectal cancer 3–5. Deng et al. reported a 26% increase in the incidence of colorectal cancer 6. Consistent with these results, Jiang et al. also described an increased incidence of colorectal cancer in a systematic review of 41 cohort studies 7. Previous studies have shown that the increase of glycolytic enzyme activity promotes colorectal cancer in diabetic rats 8. In diabetic rats, the activities of HK and PK are increased, whereas Pyruvate dehydrogenase (PDH) activity are decreased. The abnormal expression of the enzymes involved in glucose metabolism suggests that glycolysis is primarily responsible for glucose metabolism in diabetic patients 9. This phenomenon is known as the Warburg effect 10. The high rate of glycolysis provides energy for cell growth and contributes to the accumulation of glycolysis intermediates for the synthesis of nucleic acids, amino acids, and phospholipids, thereby promoting the rapid proliferation of cells 11. Furthermore, this phenomenon could provides an advantage for tumor proliferative due to the acidic microenvironment that lactic acid and hydrogen ions were generated via glycolysis 9.

Metformin (MET) is a biguanide class oral antidiabetic agent and one of only two oral antidiabetics described in the World Health Organization Model List of Essential Medicines (the other being glibenclamide). MET primarily decreases hyperglycemia through the suppression of hepatic glucose production 12. In addition, MET increases insulin sensitivity, enhances peripheral glucose uptake (by inducing the phosphorylation of GLUT4 enhancer factor), decreases insulin-induced fatty acid oxidation suppression, and decreases glucose absorption from the gastrointestinal tract 13. However, the underlying molecular mechanism of MET is incompletely understood. The inhibition of the mitochondrial respiratory chain, activation of AMP-activated protein kinase, inhibition of glucagon-induced elevation of cyclic adenosine monophosphate (cAMP), consequent activation of protein kinase A (PKA), inhibition of mitochondrial glycerophosphate dehydrogenase, and an effect on gut microbiota have been proposed as potential mechanisms 14–16.

Epidemiologic studies have shown that the treatment of diabetics with MET reduced the risk of cancer-related mortality 17. A meta-analysis of published studies showed that MET significantly decreased the risk ratio (RR) for all cancer types in comprehensive. Furthermore, except for colorectal and pancreatic cancer, MET has not been associated with any significant effect on the incidence of other cancers when analyzed separately, such as prostate and breast cancers 18. Limited evidence suggests that a molecular mechanism underlying the effects of MET might prevent the diabetes-associated complications of cancer.

In this study, diabetic rat models were induced using STZ in combination with high-fat feeding, and then induce colorectal cancer using DMH. In addition, these rats were gavaged with MET to determine the chemopreventive effects on tumorigenesis of colorectal cancers in diabetic rats.

## Materials and Methods

### Reagents

Rabbit polyclonal antibodies against PKM2 were purchased from Bioworld Technology Co. Ltd. (Nanjing, China). The rabbit polyclonal antibody raised against Isocitrate dehydrogenase 1 (IDH1) was purchased from Santa Cruz Biotechnology, Inc. (Dallas, Texas, USA). STZ, DMH and MET were purchased from Sigma (St. Louis, MO).

### Animals

One hundred male SD rats (approximately 170 g) were purchased from the Beijing HFK Bioscience Co. Ltd (Beijing, China). The rats were randomly divided into five groups with 20 per group: (1) Negative; (2) STZ; (3) STZ + DMH; (4) STZ + MET; (5) STZ + DMH + MET. The animals were acclimated for 1 week. Subsequently, except those of the Negative group, all rats were provided a high-fat diet. The body weight of the rats was measured once a week. All rats were housed in standard polypropylene cages (4 rats/cage) and maintained under standardized conditions (22 ± 3°C, humidity 50 ± 10%, 12-h light/12-h dark) with free access to food pellets and tap water. All studies were performed with the approval of the animal experimental ethics review committee of Shandong University School of Medicine.

### Development of diabetes and colorectal cancer

Except those of the Negative group, all rats were injected (i.p.) with a low dose of STZ (35 mg/kg) after 2 months of high-fat dietary manipulation. The rats in the Negative group were injected with citric acid buffer. Blood samples were drawn immediately before and 1 week after injection of STZ or its vehicle from the caudal vein after fasting for 24 h. Fasting blood glucose (FBG) levels were measured by the glucose oxidase method (GOD, Applygen Technologies Inc., Beijing, China). The serum concentrations of triglyceride (TG), total cholesterol (TC), low-density lipoprotein cholesterol (LDL-C), and high density lipoprotein cholesterol (HDL-C) were determined using an automated biochemical analyzer (Toshiba-40FR, Tokyo, Japan). The serum insulin (INS) concentrations were measured using enzyme-linked immunosorbent assay (ELISA) kits according to the manufacturer’s protocol (Cusabio, Wuhan Huamei Biotech Co. Ltd., Wuhan, China). Two weeks after the injection of STZ, the rats in the STZ + DMH and STZ + DMH + MET groups were injected (i.p.) with DMH (25 mg/kg) once a week for 12 weeks. The remaining rats were injected with 0.9% NaCl. In addition, the rats in the MET treatment groups (STZ + MET and STZ + DMH + MET) received a daily gavage with MET (MET was dissolved in double distilled water). A typical human treatment dose of MET is 1000–2500 mg. 150 mg/kg was used in the present study. This dosage was translated from human equivalent dose according to Reagan-Shaw formula 19:

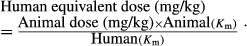


Thus, the *K*_m_ for a 60 kg human adult equals 37, whereas that for a 250 g rat equals 7.

### Identification of ACF

As an intermediate biological evaluation index, ACF were selected to detect the pathogenesis of colorectal cancer 20,21. One week after the last injection of DMH, 5 rats from each group were sacrificed to observe the formation of ACF. The colon tissues were separated and washed with 0.01 mol/L Phosphate buffer solution (PBS) briefly. Then fix the tissues in 4% paraformaldehyde for 24 h. To count the number of ACF, the tissues were stained with 0.5% methylene blue solution for 10 min. The total number of ACF and the number of foci containing a different number of crypts in the whole colon tissues were counted respectively under a light microscope (40×).

### Incidence, number, and volume of tumor

At the end of this study, the remaining rats were sacrificed. Separate the colon tissues and detect the formation of tumors. Calculate the tumor incidence, average number of tumors and tumor volume. Subsequently, the colon tissues were divided into two parts for the detection of enzymatic activity and pathology analysis respectively.

### Histological assay and PCNA staining

Embedded the tissues in paraffin and cut into 4-*μ*m sections on histology slides and subjected to HE staining and PCNA immunostaining. To determine the proliferative index (PI) of colon tissues, five high power visual fields were counted. Brown-yellow stained nuclei were considered positive. The percentage of PCNA positive nuclei among the total number of cells counted mean the PI of colon tissues.

### Immunohistochemical analysis of enzyme expression

Deparaffinating the thick sections and then heated in 0.01 mol/L citrate buffer solution (pH = 6) for 15 min for antigen retrieval. The sections were incubated with rabbit polyclonal antibodies against PKM2 and a rabbit polyclonal antibody raised against IDH1 at 4°C for 12 h. In addition, PBS and nonimmune rabbit IgG were used as controls respectively. After conjugation with the streptavidin biotin peroxidase complex, the tissues were stainined with diaminobenzidine (DAB) and then counterstained with hematoxylin. Subsequently, the tissue sections were detected using a light microscope. The percentage of DAB-stained area (tumor-positive area) were analyzed using Image J software (Bethesda, MD).

### Western blot analysis of colon tissue

At the same time, separate the frozen colon tissues (0.1 g) from each group and homogenized with RIPA using an electric homogenate machine. Centrifuged at 12,000*g* for 10 min to collect the protein and Western blotting was performed. The blots were respectively incubated with primary antibodies against PCNA, PKM2, and IDH1 at 4°C for 12 h, followed by incubation with the appropriate peroxidase-conjugated secondary antibodies. *β*-Actin served as an internal control. The immunocomplexes were visualized using an enhanced chemiluminescence detection system (Millipore, Billerica, Massachusetts, USA), followed by exposure. The gray value was analyzed using the Image J software.

### Assay of enzyme activity

Normal colon tissues, colon tumor tissues (intratumor), and tissues adjacent to tumors (peritumor) were separated, respectively, to assay the enzyme activity. Homogenized the frozen tissues (0.1 g) with 0.9 mL of normal saline using an electric homogenate machine. The entire process of grinding was performed on ice to preserve the enzymatic activity. The activities of HK and PDH were measured with Assay Kits for the detection of HK (Nanjing Jiancheng Bioengineering Institute, Nanjing, China) and the PDH Activity Colorimetric Assay Kit (Biovision Inc., San Francisco, California, USA) according to the manufacturer’s instructions.

### Cell growth and enzyme expression

Human colon cancer cells LoVo and HT-29 (American Type Culture Collection, Manassas, Virginia, USA) were authenticated in April 2014 and cultured in Dulbecoo minimum essential medium (DMEM) high-glucose medium (HyClone) supplemented with 10% fetal bovine serum (Gibco, Grand Island, New York, USA). The cells were maintained in 5% CO_2_ at 37°C until reaching 60–80% confluence and subsequently treated with MET at different concentrations (0, 5, 10, 20, and 40 mmol/L) for 48 h. The highest inhibition concentration (40 mmol/L) was selected for the treatment at different times (0, 24, and 48 h). The growth of cells was measured using the MTT (3-(4,5-dimethylthialzol-2-yl)-2,5-diphenytetrazolium bromide) assay. The protein extracts were prepared from cells treated with MET and Western blotting was performed. The blots were respectively incubated with primary antibodies against PKM2 and IDH1 as described above. At the same time, treated these two cell lines with metformin as described above. Centrifuged at 500 rpm for 5 min to collect the cells and then homogenized with 0.9 mL of normal saline using an electric homogenate machine. The activities of HK and PDH were measured according to the manufacturer’s instructions.

### Statistical analyses

All comparative data are expressed as the means ± SEM and all *P*-values are two-tailed. For parameters with Gaussian distribution, unpaired Student’s *t*-test was used. The tumor incidence was compared using the Chi square test. *P* < 0.05 was considered statistically significant. The data analysis was performed using the SPSS 18 (SPSS Software, SPSS Inc., Chicago, Illinois, USA).

## Results

### Physical parameters

**Figure 1:**
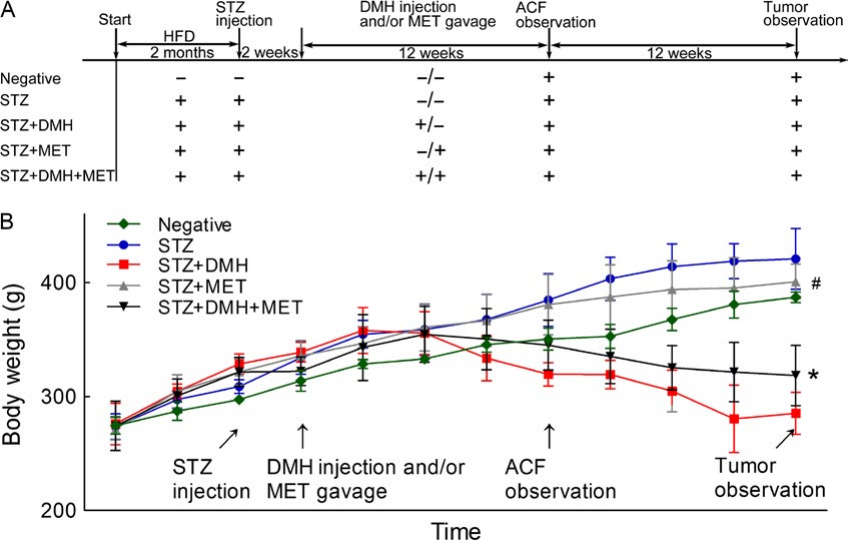
(A) Diagrammatic representation of the experiment and the animal treatment procedures. Streptozotocin (STZ) was administered at 35 mg/kg through i.p. injection, and dimethylhydrazine (DMH) was administered at 25 mg/kg per week through i.p. injection. Metformin (MET) was administered orally at 150 mg/kg per day. Five rats from each group were sacrificed at the point of Aberrant crypt foci (ACF) observation. (B) The effect of MET treatment on body weight throughout the entire experiment. The values are expressed as the means ± SEM. The parameters of body weight between two groups were analyzed using unpaired Student’s *t*-test at *P* < 0.05. At the end of the experiment, *P < 0.05 versus STZ + DMH group. ^#^*P* < 0.05 versus STZ group.

The time course of the experiment and the change in the body weight of all rats are shown in Figure1. During diabetes induction, one rat from each of the STZ and STZ + MET groups died, likely reflecting the unexpected increase in the blood glucose level. Two weeks prior to tumor observation, 2 rats in the STZ + DMH group also died may due to a severe weight loss.

Over the two-month experimental period, the weight of the rats fed a high-fat diet increased faster than those in the Negative group. After repeated injection with DMH, the weight of the rats in the STZ + DMH and STZ + DMH + MET groups decreased. In addition, the weight of the rats in the STZ + DMH group decreased faster than those in the STZ + DMH + MET group. This mean that MET treatment reduced the DMH-induced weight decrease. Whereas in the other three groups, the weight of the rats continued to increase. The weight of the rats injected with STZ rapidly increased compared with those in the Negative group. In addition, the MET gavage decreased the weight of these rats compared with those in the STZ group.

### Serological analysis

In order to detect whether the diabetic models were induced successfully, blood biochemical indices were measured immediately before and 1 week after the injection of vehicle or STZ (Table1). Before the injection of STZ, FBG as well as TG, LDL-C and INS were all significantly increased in HFD-fed rats (*P* < 0.05, respectively). However, there was a reduction of HDL-C (*P* < 0.05). Injection of STZ resulted in a significant increase of FBG, TG, TC, and LDL-C associated with a significant reduction of HDL-C. In addition, although the injection of STZ produced a reduction of INS level in HFD-fed rats (*P* < 0.05), the level of INS was still considerably higher than that of in NPD-fed rats (*P* < 0.05).

**Table 1:** Level of blood biochemical indexs

	NPD-fed (negative group)		HFD-fed (STZ, STZ + DMH, STZ + MET, STZ + DMH + MET group)	
	Before	After	Before	After
FBG (mmol/L)	4.48 ± 0.08	4.58 ± 0.06	5.94 ± 0.16*	13.98 ± 0.28**#
TG (mmol/L)	0.59 ± 0.03	0.58 ± 0.03	0.88 ± 0.09*	5.53 ± 0.43**#
TC (mmol/L)	1.20 ± 0.04	1.24 ± 0.09	1.85 ± 0.12*	3.90 ± 0.24**#
HDL-C (mmol/L)	0.48 ± 0.03	0.47 ± 0.02	0.38 ± 0.02*	0.21 ± 0.02**#
LDL-C (mmol/L)	0.80 ± 0.07	0.88 ± 0.04	1.01 ± 0.06*	1.50 ± 0.07**#
INS (*μ*IU/mL)	27.16 ± 1.65	27.39 ± 1.69	82.36 ± 3.70**	71.05 ± 3.57**#

Values are mean ± SEM. FBG, fasting blood glucose; TG, triglyceride; TC, total cholesterol; HDL-C, high-density lipoprotein cholesterol; LDL-C, low-density lipoprotein cholesterol; INS, insulin.

* *P *< 0.05 versus before and after of NPD groups.

** *P *< 0.01 versus before and after of NPD groups.

# *P *< 0.01 versus before of HFD groups.

### ACF observation

**Figure 2:**
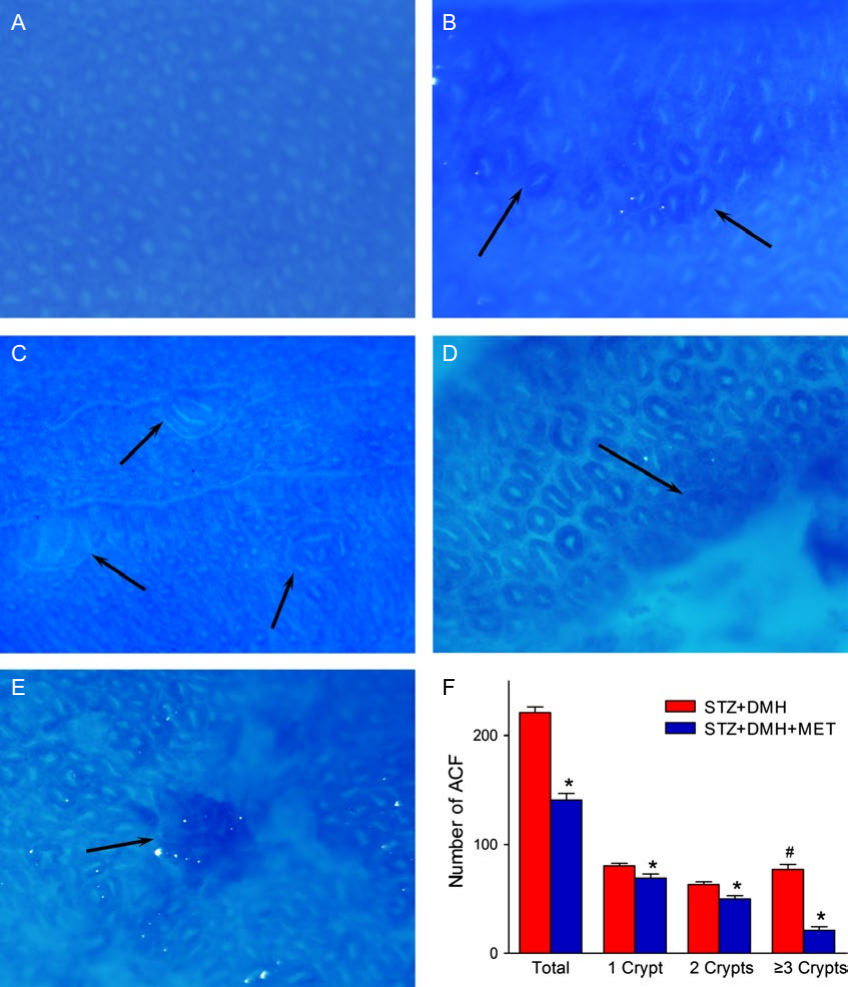
Formation of Aberrant crypt foci (ACF) induced by dimethylhydrazine (DMH) in the rat colon. (A) Normal crypt foci (40×). (B) ACF formed by 1 aberrant crypts (40×). (C) ACF formed by 2 aberrant crypts (40×). (D) ACF formed by ≥3 aberrant crypts (40×). (E) tumor-like tissue (40×). (F) Bar chart demonstrating the number of ACF and the foci containing different crypts. *P < 0.05 versus Streptozotocin (STZ)+DMH group. ^#^*P* < 0.05 versus the number of foci containing 2 crypt in STZ+DMH group.

One week after the last DMH injection, five rats from each group were sacrificed to identify the formation of ACF. ACF were detected in the DMH-induced groups (STZ + DMH and STZ + DMH + MET) but not in the other groups (Fig.2). As shown in Figure2F, the total number of ACF in the STZ + DMH group was significantly increased when compared with that in the STZ + DMH + MET group (*P *< 0.05). In addition, the number of foci containing different crypts were significantly increased (*P *< 0.05 respectively). This mean MET treatment prevents the formation of ACF. Furthermore, the number of foci containing ≥3 crypts was even higher than those containing 2 crypts in the STZ + DMH group (*P *< 0.05).

### Incidence, number, and volume of colonic tumors

All of the remaining rats were sacrificed at the end of the experiment. Tumor-bearing rats mean the rats that tumors were visualized in general through gross examination in colon tissue. Tumor incidence, average number of tumors, and tumor volume were calculated using the following formulas 22:

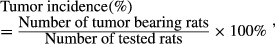


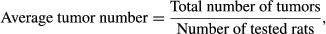


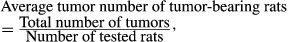


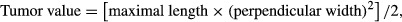


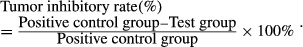


As shown in Table2, a total of 11 rats in the STZ + DMH group and nine rats in the STZ + DMH + MET group showed tumor formation in the colon tissues. In contrast, no tumors were detected in the other three groups. MET treatment reduced the incidence of tumors in the STZ + DMH + MET group when compared with the STZ + DMH group, but there was no statistical significance (*P *= 0.15). In addition, MET treatment reduced the volume, number of tumors when compared with those in the STZ + DMH group (*P *< 0.05 respectively). The volume of the tumors in the STZ + DMH group ranged from 4 to 366 mm^3^, whereas the volume of the biggest tumor in the STZ + DMH + MET group was only 171.5 mm^3^. In addition, in the STZ + DMH group, one tumor was detected in the head of one rat and another tumor was detected in the lung of another rat. These results indicated that MET treatment inhibited the incidence of tumors.

**Table 2:** Incidence, number, and volume of tumors

	Group	
	STZ + DMH	STZ + DMH + MET
*N*	13	15
Tumor-bearing rats	11	9
Tumor incidence (%)	84.62	60.00
Number of tumors	40	20
Average tumor number	3.1 ± 0.45	1.3 ± 0.37*
Average tumor number of tumor-bearing rats	3.6 ± 0.28	2.2 ± 0.36*
Volume of tumors (mm^3^)	94.8 ± 19.77	30.9 ± 8.19*
Inhibition of colorectal tumor (%)		29.08

The remaining rats were all sacrificed at the end of the experiment. Tumor-bearing rats indicate that the tumors were visualized in general by gross examination in colon tissue. Values are expressed as mean ± SEM. The parameters of tumor incidence was analyzed using Chi square test at *P* < 0.05. The average tumor number, average tumor number of tumor-bearing rats and volume of tumors were analyzed using unpaired student’s *t-*test at *P* < 0.05.

* *P *< 0.05 versus STZ + DMH group.

### Proliferation index of colorectal tumor cells

**Figure 3:**
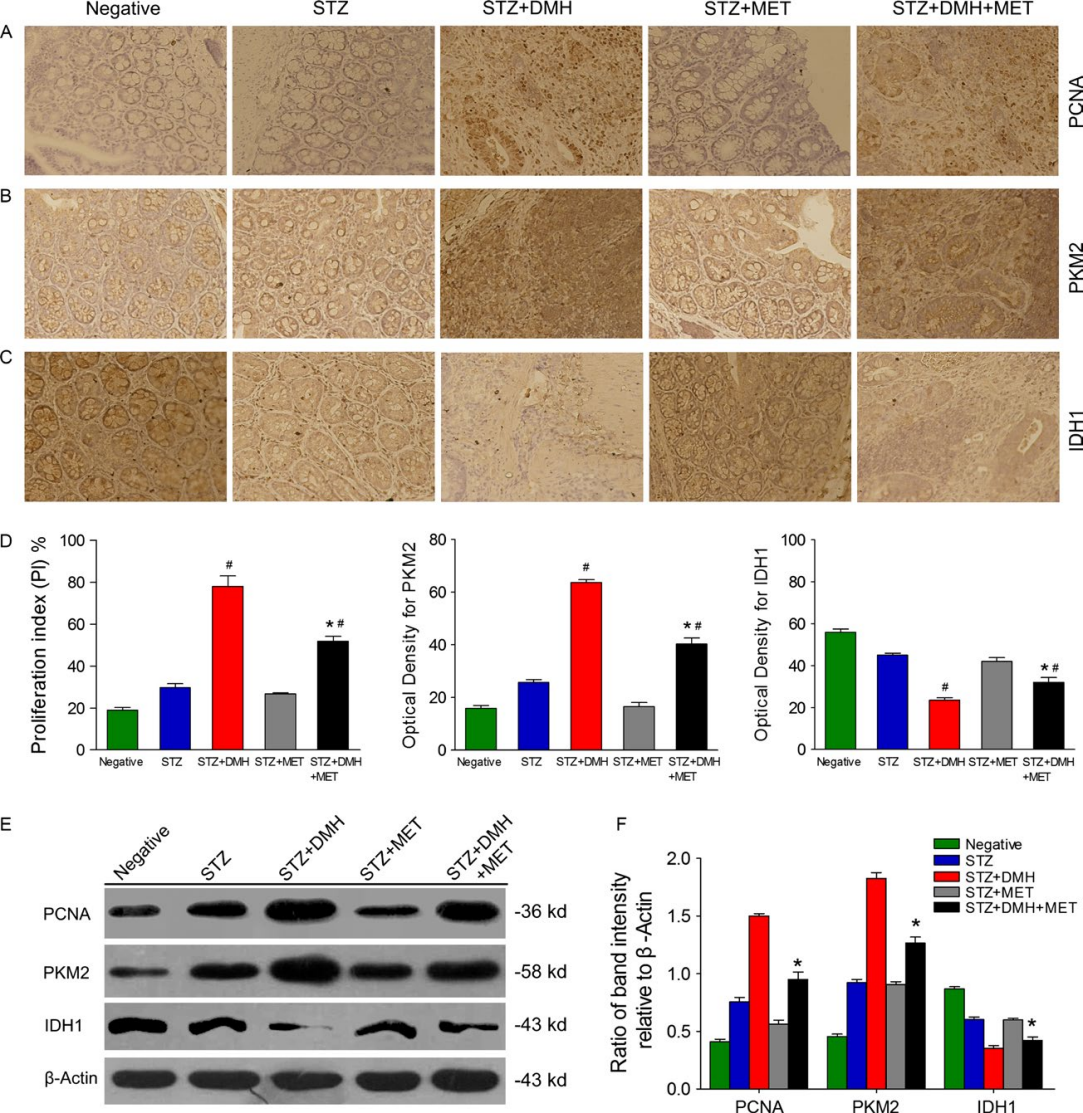
(A) Proliferating cell nuclear antigen (PCNA) immunostaining in colorectal tissues (microscope setting ×20). The brown-yellow stained nuclei were regarded to be PCNA positive. Proliferative index (PI) was expressed as the percentage of PCNA-positive nuclei among the total number of cells counted. (B) Photomicrographs and effect of different pharmacological treatments on the expression of PKM2 in the colorectal tissue. (C) A series of IHC-stained images and the effects of different pharmacological treatments on the expression of IDH1 in the colorectal tissue. (D) Bar chart demonstrating the optical density of the immunostaining for PCNA, PKM2 and IDH1 in colorectal tissues. (E) Western blot analysis of PCNA, PKM2 and IDH1 expression in colon tissues. (F) Bar chart demonstrating the ratio of band intensity relative to *β*-Actin. The values are expressed as the means ± SEM analyzed using unpaired Student’s *t*-test at *P* < 0.05. *P < 0.05 versus Negative group. ^#^*P* < 0.05 versus STZ + DMH group.

The expression of PCNA were shown in Figure3A. In DMH-induced colon tumor tissues PCNA was strongly expressed, particularly in the STZ + DMH group. These data suggest that DMH treatment significantly promoted cell proliferation, which was inhibited through MET. Indeed, treatment with MET inhibited cell proliferation as the PI was considerably reduced in the STZ + DMH + MET group compared with that in the STZ + DMH group (51.8 ± 2.32 vs. 77.9 ± 5.05, *P *< 0.05). In addition, the induction of diabetes increased cell proliferation. However, there was no significant difference between the STZ group and the STZ + MET group (29.7 ± 1.82 vs. 26.7 ± 0.57, *P* = 0.149).

### Expression of enzymes in colon tissues

Photomicrographs of the immunohistochemical staining and Western blot for PKM2 and IDH1 are shown in Figures3. The sections incubated with PBS or nonimmune rabbit IgG were negative. Statistical analysis revealed that the injection of DMH increased immunoreactivity for PKM2. In addition, MET treatment reduced the expression of PKM2 compared with the STZ + DMH group (40.36 ± 2.25 vs. 63.67 ± 1.03, *P *< 0.05). Furthermore, the immunoreactivity in the STZ group was increased compared with the Negative group (25.71 ± 1.12 vs. 15.92 ± 1.08, *P *< 0.05) but decreased after MET treatment (25.71 ± 1.12 vs. 16.55 ± 1.58, *P *< 0.05). The results of Western blot were in good agreement with the immunohistochemical results.

The injection of DMH reduced the immunoreactivity for IDH1, and IDH1 activity was increased with MET treatment compared with that in the STZ + DMH group (32.02 ± 2.30 vs. 23.58 ± 1.15, *P *< 0.05). Moreover, the immunoreactivity for IDH1 in the STZ and STZ + MET groups was decreased compared with that in the Negative group (41.97 ± 1.82 or 44.99 ± 0.92 vs. 55.97 ± 1.46, *P *< 0.05), and no statistical significance was observed between these two groups (41.97 ± 1.82 vs. 44.99 ± 0.92, *P *= 0.18). The results of Western blot consistently with the immunohistochemical.

### Analysis of enzyme activity in colonic tissue

No tumors were detected in the Negative, STZ or STZ + MET groups; therefore, separate the tissue samples randomly. In the STZ + DMH and STZ + DMH + MET groups, the tissue samples were separated from the peritumoral and the intratumoral regions respectively.

**Figure 4:**
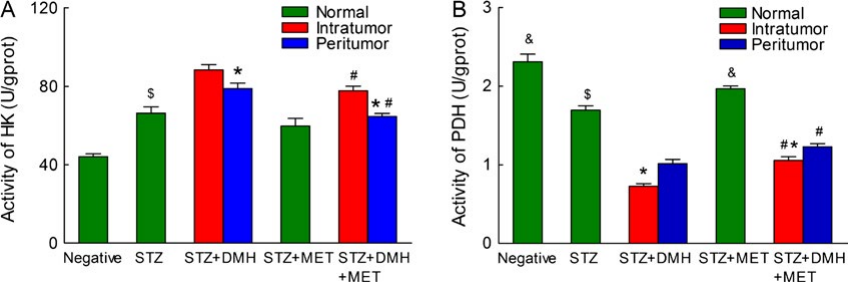
Bar charts demonstrating the activity of HK and PDH in colon tissues. No tumor formation was detected in the Negative, Streptozotocin (STZ) and STZ + MET groups, thus the tissue samples were dissected at random. The samples were collected from the intratumoral and the peritumoral regions in the STZ + DMH group and the STZ + DMH + MET group, respectively. The results are expressed as the means ± SEM analyzed using unpaired Student’s *t*-test at *P* < 0.05. (A) Analysis of HK. *P < 0.05 versus intratumor. ^#^*P* < 0.05 versus STZ+DMH group. ^$^*P* < 0.05 versus the DMH-induced in the intratumor. (B) Analysis of PDH. *P < 0.05 versus peritumor. ^#^*P* < 0.05 versus STZ+DMH group. ^$^*P* < 0.05 versus the DMH-induced in the intratumor or peritumor. ^&^*P* < 0.05 versus the STZ group.

The activity of HK were shown in Figure4A. Due to the injection of DMH, HK activity were increased in both intratumoral and peritumoral tissues, and the activity of HK in intratumoral tissues was higher than that in peritumoral tissues (*P *< 0.05, respectively). MET treatment reduced the HK activity in both intratumoral and peritumoral tissues (88.32 ± 2.68 vs. 77.67 ± 2.42 and 78.78 ± 2.79 vs. 64.60 ± 1.51, *P *< 0.05, respectively). Moreover, the HK activity in normal tissues in the STZ + MET group was decreased after MET treatment compared with that in the STZ group (57.70 ± 3.90 vs. 66.26 ± 3.24, *P *< 0.05).

Figure4B shows the activity of PDH. A significant reduction in PDH activity was observed in the DMH-induced rats compared with that in those of the Negative and STZ groups (*P *< 0.05 respectively). In addition, the PDH activity was significantly decreased in intratumoral tissues than that in the peritumoral tissues (*P *< 0.05 respectively). Moreover, following MET treatment, the PDH activity in both intratumoral and peritumoral tissues was increased compared with that in the STZ + DMH group (1.06 ± 0.05 vs. 0.73 ± 0.04 and 1.23 ± 0.04 vs. 1.02 ± 0.05, *P *< 0.05 respectively).

### Influence of MET on cell growth and enzyme expression

**Figure 5:**
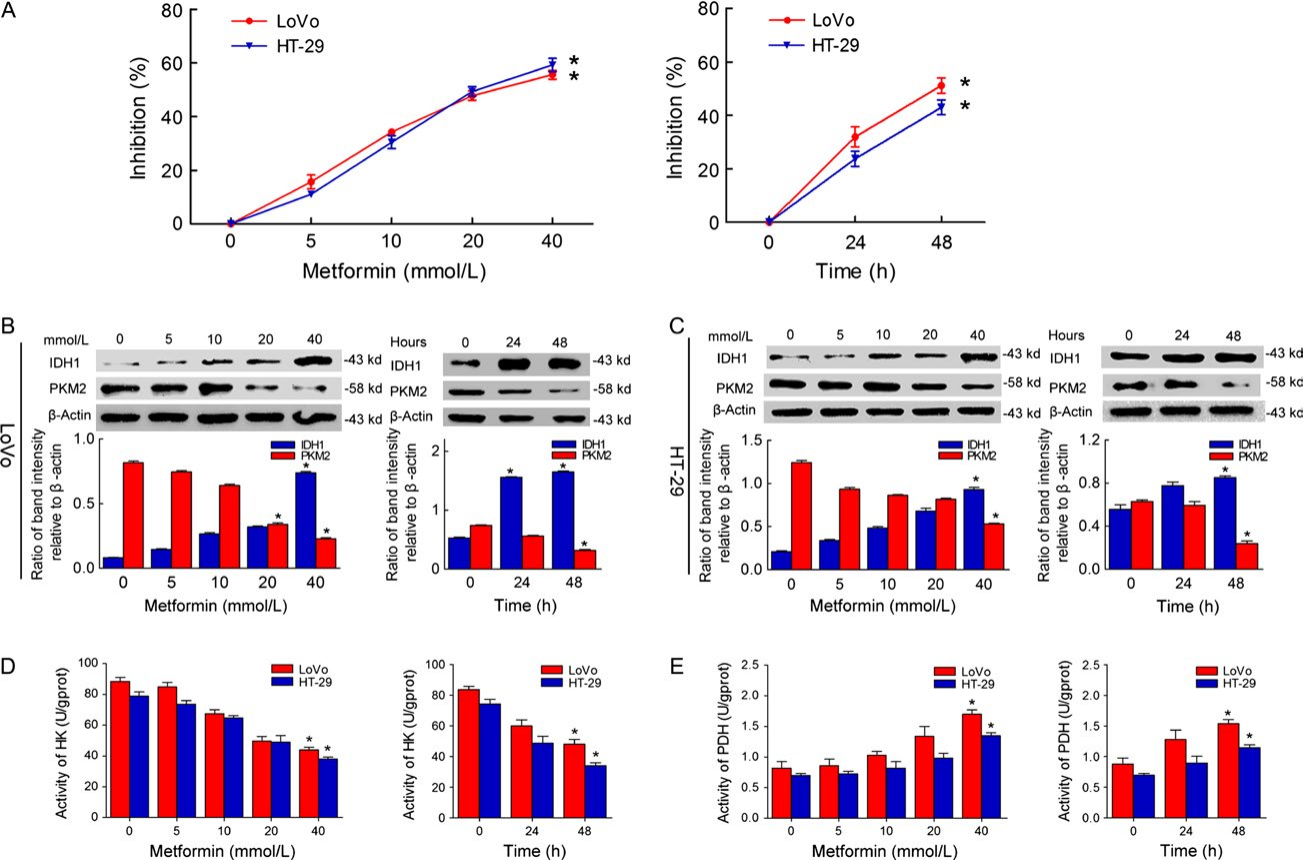
Effect of metformin (MET) treatments on the growth and expression of PKM2 and IDH1 and the activity of HK and PDH in LoVo and HT-29 cells. (A) The effect of MET treatment at different concentrations and for different times on the growth of cells, *P < 0.05 versus 0 point; (B) The effect of MET treatment at different concentrations and for different times on the expression of PKM2 and IDH1 in LoVo cells, *P < 0.05 versus 0 point respectively; (C) The effect of MET treatment at different concentrations and for different times on the expression of PKM2 and IDH1 in HT-29 cells, *P < 0.05 versus 0 point respectively. (D) The effect of MET treatment at different concentrations and for different times on the activity of HK in LoVo and HT-29, *P < 0.05 versus 0 point respectively. (E) The effect of MET treatment at different concentrations and for different times on the activity of PDH in LoVo and HT-29, *P < 0.05 versus 0 point respectively. The relative grey value indicates the odds ratio of target protein/*β*-Actin. Inhibition of cell growth = (OD (0) – OD (x))/OD (0).

Following MET treatment at different concentrations and for different times, the inhibition of cell growth was measured using the MTT assay. MTT assay revealed that MET treatment resulted in dose- and time-dependent growth inhibition. Inhibition effect increased with increasing MET concentration and incubation time (Fig.5A). Figure5B and C shows the expression of PKM2 and IDH1 in LoVo and HT-29 cells after MET treatment at different concentrations and for different times. IDH1 expression increased, whereas PKM2 expression decreased with increasing MET concentration and treatment times. The activity of HK and PDH in LoVo and HT-29 after the treatment of MET were shown in Figure5D and E. With the increasing MET concentration and treatment times, the activity of HK gradually decreased. In contrast, the activity of PDH gradually increased.

## Discussion

The main objective of this study was to determine whether MET could decrease the risk of DMH-induced colorectal carcinogenesis in type 2 diabetic rats. In cancer cells, the increased aerobic glycolysis and enhanced lactate production was known as Warburg effect. Previous study have indicated that diabetes promote DMH-induced colorectal cancer by increasing Warburg effect. The second objective was to determine whether the inhibition on Warburg effect play a role in this process. Therefore, the expression of PK and IDH and activities of HK and PDH in colonic tissues and coloncancer cells were measured after metformin treatment. Thus, type 2 diabetic rat models, closely reflecting the metabolic characteristics of human type 2 diabetes patients, were induced. Subsequently, colorectal carcinogenesis were induced using DMH, at the same time treated with MET.

First, the type 2 diabetic rats were induced using a low dose of STZ in combination with high-fat feeding. Srinivasan et al. reported that rats induced in this way adequately simulates the human syndrome and is suitable for testing anti-diabetic agents for the treatment of type 2 diabetes 23,24. After the injection of STZ, the level of the blood biochemical indices in the HFD-fed groups were all significantly increased, except for HDL-C and INS. The reduction of INS may due to destruction of pancreatic beta cells by injection of STZ, but the level of INS was still higher than that of the NPD-fed rats (Table1). These data suggest that the diabetic models were induced successfully. Subsequently, the rats were injected with DMH to induce colorectal carcinogenesis as previously described 25.

Previous studies have shown a close link between diabetes and colorectal cancer whereby diabetes could accelerate the incidence of colorectal cancer. The dysfunction of energy metabolism pathways has been implicated in the occurrence and development of colorectal cancer, and the abnormal expression of enzymes involved in glycometabolism might also play an important role in the development of colorectal cancer 8.

MET is a biguanide oral antidiabetic agent. Recent studies have explained that MET inhibits cancer cell growth and blocks tumor growth 26–29. In the present study, colorectal cancer was induced in diabetic rats to investigate the inhibitory effects of MET. The formation of ACF and tumors, the incidence, number and volume of the tumors in colon tissues were measured as evaluation indices. In both rodents and humans, ACF are precancerous lesions in the pathogenesis of colorectal cancer induced through carcinogens 30–32. In the present study, MET treatment have inhibited the formation of ACF when compared with the STZ + DMH group. In addition, the proportion of the foci containing a different number of crypts was changed (Fig.2). In the STZ + DMH group, the proportion of foci containing ≥3 crypts was 34.87%, a value higher than that containing 2 crypts (28.68%). However, when treated with MET, this proportion was only 14.94%. No ACF were detected in the other three groups. These data indicate that MET treatment reduced the formation and occurrence of colorectal cancer. At the end of the preaent study, the incidence, number and volume of tumors in STZ + DMH + MET group were reduced after MET treatment (Table2). These data indicate that MET retarded the progression of existing precancerous lesions and the growth of tumors in the colon tissues.

PCNA is a DNA clamp that acts as a processivity factor for DNA polymerase *δ* in eukaryotic cells and is essential for replication. The expression of PCNA reflects the proliferation activity of cellular and is a reliable index for evaluating tumor cell proliferation 33. The results obtained in the present study shown that the expression of PCNA in all DMH-induced tumor tissues were higher when compared with the normal tissue and that MET treatment apparently inhibited the proliferation of colon cancer cells when compared with the STZ + DMH group (Fig.3). These results indicated that MET exerts anti-tumor effects through the inhibition of cancer cell proliferation.

To determine the energy metabolism pathways dysfunction that involved in glycometabolism, we assayed the alteration of HK and PDH activities in intratumoral tissues, peritumoral tissues and normal tissues. The results showed that HK activity was significantly increased in intratumoral tissues and peritumoral tissues than in normal tissues. In contrast, PDH activity was reduced. With MET treatment, the alteration of HK and PDH activities were reduced compared with those in the STZ + DMH group (Fig.4). These data demonstrate that MET might decrease the rate of glycolysis and reduce the incidence of colorectal cancer.

PKM2 is an isoenzyme of the glycolytic enzyme PK. The expression of different PK isoenzymes are depending on the different metabolic functions of the tissues. PKM2 is expressed in some differentiated tissues and in all cells with a high rate of nucleic acid synthesis, particularly tumor cells 34–36. Initially, a switch from PKM1 to PKM2 expression during tumorigenesis was discussed. In the present study, PKM2 was highly expressed in the colonic tissues of diabetic rats, consequently increasing the production of glycolytic intermediates and providing an acidic microenvironment for tumor growth. After the injection of DMH, the expression of PKM2 in colonic tissues was increased. Combined with MET treatment, PKM2 expression in the STZ + DMH + MET group was decreased, but the expression of this enzyme was still higher than that in normal tissues (Fig.3). It has been reported that the genetic manipulation of cancer cells to produce PKM1 instead of PKM2 reverses the Warburg effect and potentially reduces the growth rate of these modified cancer cells 37. The data obtained in the present study indicate that MET could reduce the incidence of DMH-induced colorectal cancer through a reduction of PKM2 expression. Consistently, the expression of PKM2 in colon cancer cells LoVo and HT-29 decreased with increasing MET concentration and processing time (Fig.5). Several studies have shown that PKM2 occurs in both a tetrameric form and a dimeric form. The tetrameric form of PKM2 has a high affinity for the phosphoenolpyruvate, whereas the dimeric form of PKM2 has a low affinity to phosphoenolpyruvate and is nearly inactive at physiological phosphoenolpyruvate concentrations. When PKM2 is primarily in dimeric form, which is the case in tumor cells, all glycolytic intermediates above PK accumulate and are channeled into synthetic processes 37,38. Intermediates are important building blocks required by highly proliferating cells, such as tumor cells. Due to the key position of PK in glycolysis, the PKM2 tetramer: dimer ratio determines whether glucose carbons are converted to pyruvate and lactate for energy production or channeled into synthetic processes. Therefore, further studies are needed to investigate the alteration of PKM2 conformation.

IDH1 is an isoenzyme of IDH, and as a key enzyme in the TCA cycle, this enzyme catalyzes the oxidative decarboxylation of isocitrate to produce *α*-ketoglutarate. In the present study, the expression of colonic IDH1 in DMH-induced diabetic rats was decreased. MET treatment increased colonic IDH1 expression in the STZ + DMH + MET group. It has been reported that reduced IDH1 expression is associated with decreased p53 expression and that IDH1 expression is negatively correlated with tumor metastasis 39. Robbins also demonstrated that decreased IDH1 expression might be correlated with tumor promotion 40. Li et al. showed that the levels of IDH1 mRNA were significantly decreased in cancerous tissues compared with those in paired paracancerous normal tissues. The down-regulation of IDH1 observed in colorectal cancer cells might reflect the fact that cancer cells do not prefer to use the TCA cycle for energy 41. The isoforms IDH1 and IDH2 catalyze the same reaction. Lv et al. observed that IDH2 gene expression was significantly downregulated in early stage carcinoma but upregulated in advanced stage carcinoma 42. However, Li did not observed alterations in the IDH2 mRNA expression levels between paired cancerous tissues and paracancerous normal tissues. Therefore, alterations in IDH1 and IDH2 expression might play different roles during the development of colorectal cancer. However, further studies are needed to confirm this hypothesis.

**Figure 6:**
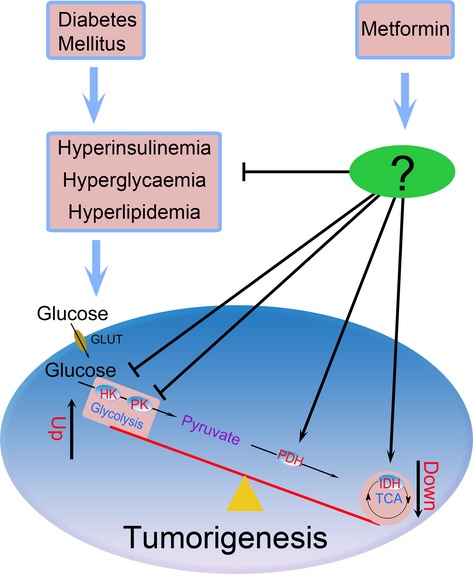
Metformin prevents dimethylhydrazine-induced colorectal cancer in diabetic rats by reversing the Warburg effect.

Taken together, the results of the present study suggested that MET significantly decreases the amount of precancerous lesions and inhibits colon carcinogenesis induced through DMH in diabetic rats. In addition, MET supplementation might exert significant and potentially beneficial effects on the suppression of colon cancer cell proliferation. MET might also reduce the imbalance between glycolysis and oxidative phosphorylation and reverse the Warburg effect (Fig.6). The inhibition of glycolysis might decrease glycolytic intermediate accumulation and retard cell proliferation. The precise anti-tumor mechanisms of MET need further investigation.

## Conflict of interest

The authors have no conflicts of interest to declare.
